# Unveiling the partners of the DRBD2-mRNP complex, an RBP in *Trypanosoma cruzi* and ortholog to the yeast SR-protein Gbp2

**DOI:** 10.1186/s12866-019-1505-8

**Published:** 2019-06-11

**Authors:** Helisa Helena Wippel, Juliane Soldi Malgarin, Alexandre Haruo Inoue, Felipe da Veiga Leprevost, Paulo Costa Carvalho, Samuel Goldenberg, Lysangela Ronalte Alves

**Affiliations:** 1 Carlos Chagas Institute-Fiocruz, Professor Algacyr Munhoz Mader, 3775, Curitiba, Paraná Brazil; 2 Molecular Biology Institute-Paraná, Curitiba, Brazil; 3 Medical Science Unit I, Department of Pathology, University of Michigan, EUA, 1301 Catherine St, Ann Arbor, MI 48109 USA

**Keywords:** RNA-binding protein, Gene expression regulation, RNA metabolism, *Trypanosoma cruzi*

## Abstract

**Background:**

RNA-binding proteins (RBPs) are well known as key factors in gene expression regulation in eukaryotes. These proteins associate with mRNAs and other proteins to form mRNP complexes that ultimately determine the fate of target transcripts in the cell. This association is usually mediated by an RNA-recognition motif (RRM). In the case of trypanosomatids, these proteins play a paramount role, as gene expression regulation is mostly posttranscriptional. Despite their relevance in the life cycle of *Trypanosoma cruzi*, the causative agent of Chagas’ disease, to date, few RBPs have been characterized in this parasite.

**Results:**

We investigated the role of DRBD2 in *T. cruzi*, an RBP with two RRM domains that is associated with cytoplasmic translational complexes. We show that DRBD2 is an ortholog of the Gbp2 in yeast, an SR-rich protein involved in mRNA quality control and export. We used an immunoprecipitation assay followed by shotgun proteomics and RNA-seq to assess the interaction partners of the DRBD2-mRNP complex in epimastigotes. The analysis identified mostly proteins involved in RNA metabolism and regulation, such as ALBA1, ALBA3, ALBA4, UBP1, UBP2, DRBD3, and PABP2. The RNA-seq results showed that most of the transcripts regulated by the DRBD2 complex mapped to hypothetical proteins related to multiple processes, such as to biosynthetic process, DNA metabolic process, protein modification, and response to stress.

**Conclusions:**

The identification of regulatory proteins in the DRBD2-mRNP complex corroborates the important role of DRBD2 in gene expression regulation in *T. cruzi*. We consider these results an important contribution to future studies regarding gene expression regulation in *T. cruzi*, especially in the field of RNA-binding proteins.

**Electronic supplementary material:**

The online version of this article (10.1186/s12866-019-1505-8) contains supplementary material, which is available to authorized users.

## Background

*Trypanosoma cruzi* is the causative agent of Chagas’ disease, which affects approximately 7 million people worldwide and is endemic in Latin America [1]. In trypanosomes, the control of gene expression occurs mainly at the posttranscriptional level through messenger RNA (mRNA) processing in the nucleus, its transport to the cytoplasm, translation, and degradation [2, 3].

*T. cruzi* is exposed to diverse biological conditions in nature because the life cycle of the parasite occurs in two different hosts (mammalian and insect) [4]. The environmental and morphological changes throughout the life cycle of *T. cruzi* lead to changes in gene expression and metabolic pathways in this organism [5]. RNA-binding proteins (RBPs) are well described as key players in gene expression regulation in eukaryotes [6]. These proteins associate with mRNAs and other proteins to form ribonucleoprotein (mRNP) complexes that ultimately determine the fate of target transcripts in the cell [7]. This association is usually mediated by the RNA-recognition motif (RRM), the most frequent domain in RBPs in eukaryotes [8–10].

Despite their importance in mRNA regulation, few RBPs have been characterized in *T. cruzi* [11–13]. Some examples are TcUBP1 and TcUBP2, two RBPs that act in the destabilization and degradation of target transcripts by binding to AU-rich elements (ARE) present in the 3′-UTRs of target mRNAs [14, 15]. TcDHH1 is a cytoplasmic DEAD box helicase involved in mRNA metabolism [16]. TcNRBD1 is associated with the translation machinery*;* conversely*,* its ortholog in *T. brucei* is involved in ribosomal RNA metabolism [11]. The zinc finger protein ZC3H39 in *T. cruzi* regulates specific sets of transcripts depending on the physiological condition of the parasite [17]; when subjected to nutritional stress, this protein binds to highly expressed mRNA transcripts, such as ribosomal proteins and cytochrome C oxidase. TcRBP9 is an RBP involved in translation regulation in *T. cruzi* [17]; TcRBP9 associates with proteins involved in RNA metabolism in epimastigotes and with several translation initiation factors in nutritionally stressed epimastigotes. RBSR1 is a recently characterized nuclear RBP [18]; this protein is an ortholog to the human splicing factor SRSF7 and regulates a set of small nucleolar RNAs and small nuclear RNAs, indicating a role in RNA processing in the nucleus.

In *T. brucei*, a considerable number of RBPs have previously been described [19, 20]. We highlight the ALBA proteins, which are RBPs associated with the translation machinery in *T. brucei* [21, 22]; ALBAs 1, 2, 3, and 4 are recruited to mRNP complexes under nutritional stress and thus are likely linked to a role in controlling translation. Interestingly, ALBAs 3 and 4 associate with DHH1 to form stress granules [21, 22]. In *Leishmania*, ALBAs 1 and 3 associate with other RBPs and ribosomal subunits to form a cytoplasmic mRNP that ultimately represses translation [23].

Here, we investigated the role of DRBD2 in *T. cruzi*, a cytoplasmic RBP with two RRM domains. We show that DRBD2 is an ortholog of Gbp2 in yeast, a shuttling serine/arginine (SR)-rich protein involved in mRNA quality control and export [24]. In *T. brucei*, previous reports have indicated that DRBD2 destabilizes its target mRNAs, as described in yeast [25]. In our study, a DRBD2 sedimentation profile on a sucrose density gradient demonstrated that the protein is present in translational complexes. With this result as motivation, we used immunoprecipitation assays followed by shotgun proteomics and RNA-seq to assess the partners of the DRBD2-mRNP complex; our analysis identified mostly proteins involved in RNA regulation and metabolism. We highlight the RBPs ALBA1 [22, 23], ALBA3 and ALBA4 [21, 22], UBP1 and UBP2 [14, 15], DRBD3 [19, 26], and the poly(A) binding protein PABP2 [27]. Interestingly, the RNA-seq analysis identified mostly transcripts mapped to hypothetical proteins. Taken together, our results help elucidate the role of DRBD2 in gene expression regulation in *T. cruzi* through its association to well-known RNA binding proteins.

## Results

### DRBD2 is an ortholog of the SR-protein Gbp2 in yeast

The results from BLAST together with those from the multiple sequence analysis provided convincing evidence that the *T. cruzi* DRBD2 (TcCLB.510755.120) protein is an ortholog of Gbp2 proteins in other organisms, such as *T. brucei* and *S. cerevisiae*, with approximately 40% similarity between the sequences. However, domain conservation and the protein sizes show that these proteins acquired - or lost - different functions during evolution (Additional file 1: Figure S1).

**Fig. 1:**
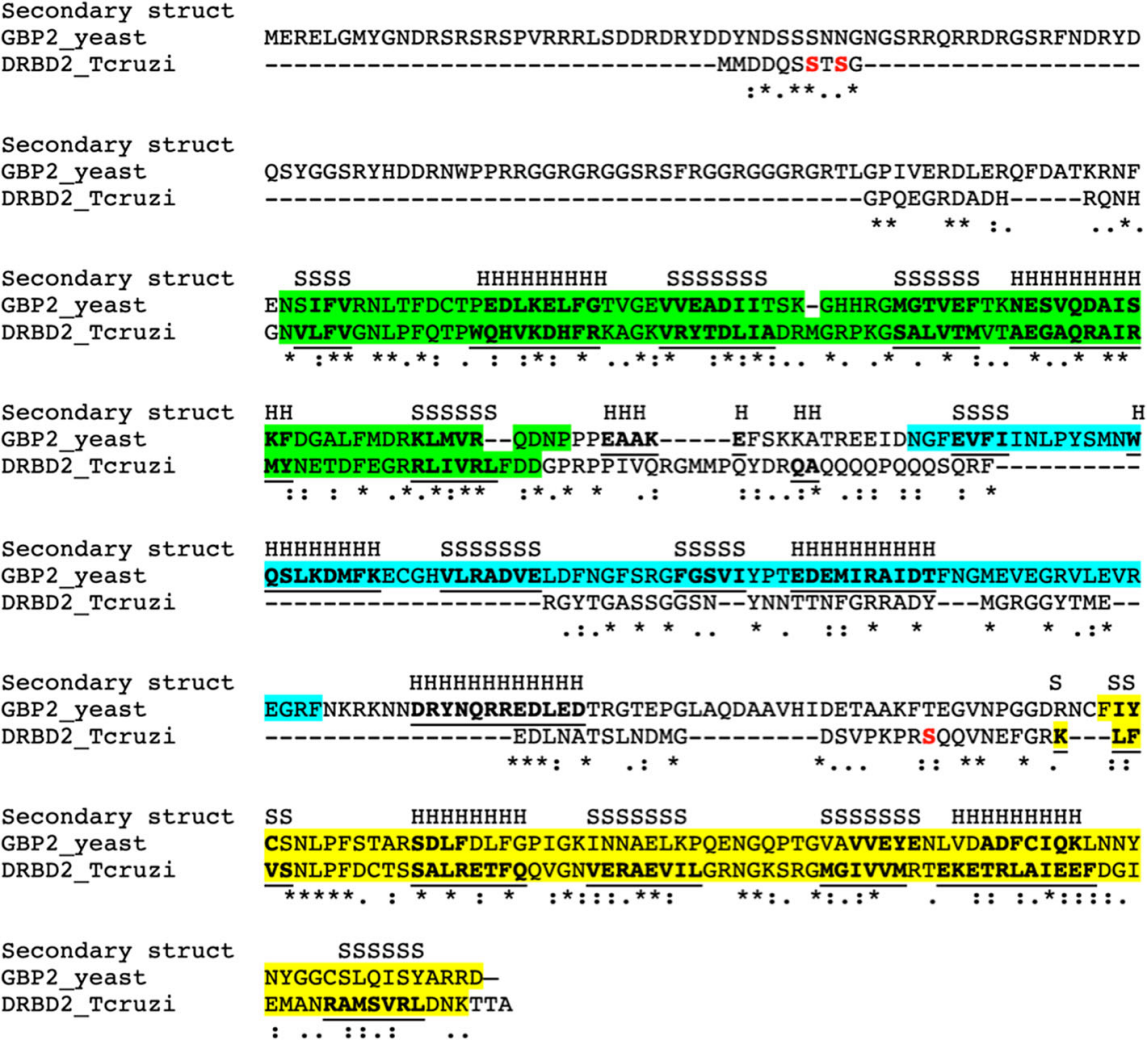
DRBD2 and yeast Gbp2 alignment and secondary structure maps. In green is the first RRM domain, in blue the second RRM domain, present only in Gbp2 and in yellow the third RRM domain. In bold and underlined the amino acids that are part of the secondary structure (S - β-sheets and H - α-helixes). The Serine residues in red are the phosphorylation sites of DRBD2. Symbols - “*” (asterisk) fully conserved amino acid residues; “:“ (colon) conservation between groups of strongly similar properties; “.” (period) conservation between groups of weakly similar properties

DRBD2 is an RBP with two RRM domains Fig. 1. Its ortholog in yeast, Gbp2, is an SR-rich protein with three RRM domains (Fig. 1) [28]. The SR-protein Gbp2 forms a mRNP complex that acts in the quality control and export of recently spliced mRNAs [24]; after mRNA transport, the protein remains associated with the transcripts, suggesting a role in translation regulation [29].

Although DRBD2 does not have SR-rich motifs as in Gbp2, DRBD2 contains several predicted phosphorylation sites in its sequence (Additional file 2: Figure S2). Phosphorylation is the main posttranslational modification responsible for nuclear transport and other cellular processes [30]. The phosphoproteome analysis of *T. cruzi* identified phosphorylated cysteine residues in DRBD2 from exponentially growing epimastigotes (Fig. 1a) [31]. Based on this result, we hypothesize that DRBD2 is involved in (i) mRNA transport, such as the Gbp2 *modus operandi* [29], and/or (ii) binding transcripts being exported from the nucleus, as DRBD2 is an RBP with two RRM domains [8].

### TAP-tagged DRBD2 is detected throughout metacyclogenesis

**Fig. 2:**
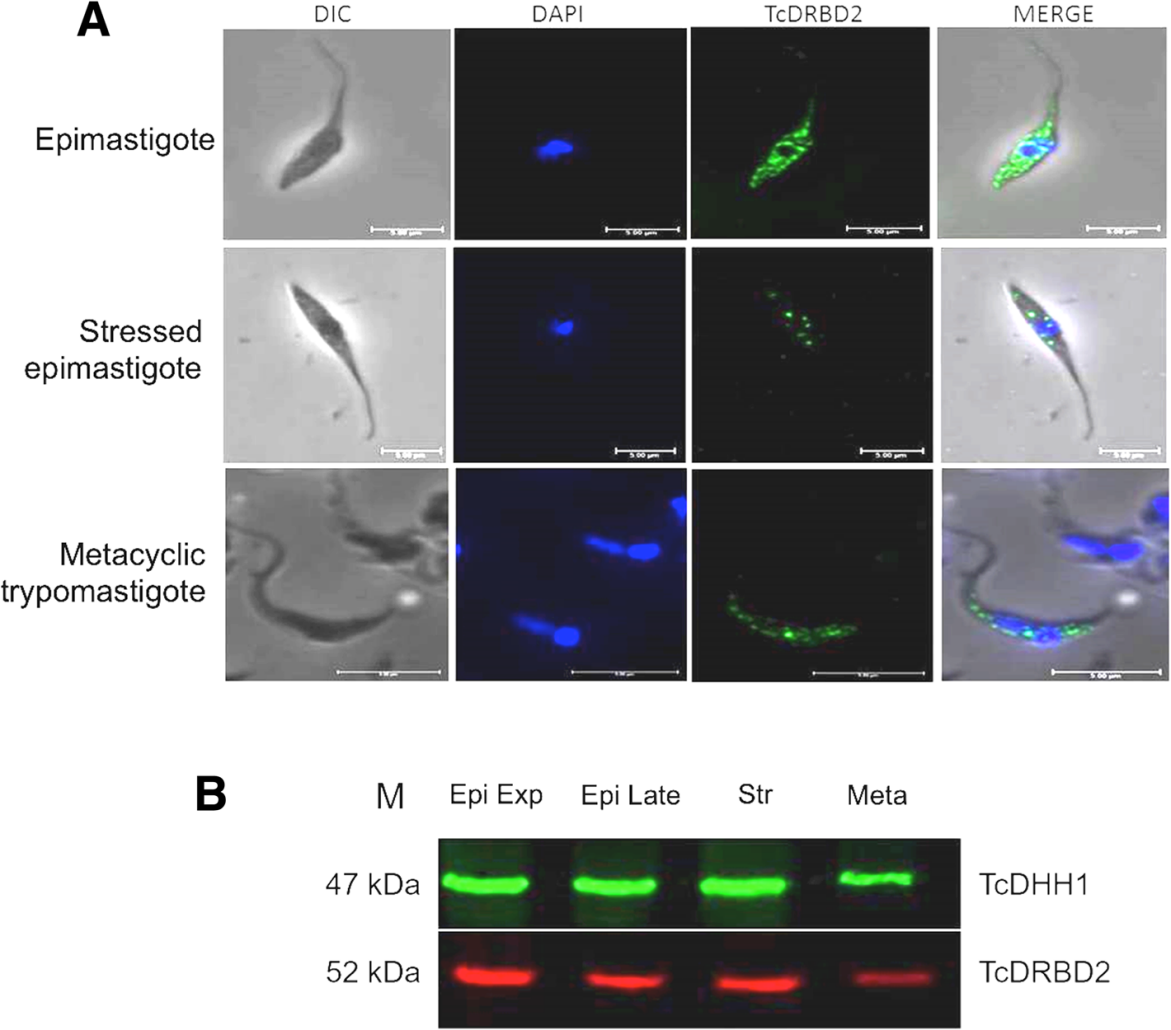
DRBD2 cellular localization and expression pattern throughout metacyclogenesis. **a** Detection of TAP-tagged DRBD2 by indirect immunofluorescence. DIC: differential interference contrast; DAPI: DNA stained with 4′,6-diamidino-2-phenylindole (DAPI); TcDRBD2: endogenous TAP-tagged DRBD2 detected by anti-protein A primary antibody (1:40,000 dilution), and secondary antibody conjugated to Alexa 488 (1:400 dilution); Merge: merge between TcDRBD2 and DAPI images. **b** Detection of DRBD2 throughout metacyclogenesis by immunoblotting. Epi exp. - Epimastigotes in exponential growth; Epi late - Epimastigotes in late exponential growth; Str - Epimastigotes subjected to nutritional stress; Meta - Metacyclic trypomastigotes. Estimated molecular weight of TAP-tagged DRBD2: 52 kDa; TcDHH1 (control): 47 kDa. M: BenchMark™ Protein Ladder (Thermo Fisher Scientific). Bound antibodies were detected by the Odyssey® imager. Approximately 5,0 × 10^6^ parasites per well were used

TAP-tagged DRBD2 is detected throughout the metacyclogenesis process (Fig. 2a and b). It is a cytoplasmic protein, slightly concentrated around the nucleus in unstressed epimastigotes and metacyclic trypomastigotes (Fig. 2a). This pattern of cellular localization would corroborate the hypothesis raised above, that DRBD2 could have a role in the nuclear transport of its target transcripts. In stressed parasites, however, DRBD2 altered its localization, displaying a granular formation scattered in the cytoplasm (Fig. 2a); this shift in the protein localization suggests an alteration of DRBD2 function during stress. In addition, RNA-seq data available at tritryp database, showed that the drbd2 gene is expressed in all developmental forms of the parasite (Additional file 3: Figure S3 A and B) [32–34].

### DRBD2 is present in translational complexes in unstressed epimastigotes

**Fig. 3:**
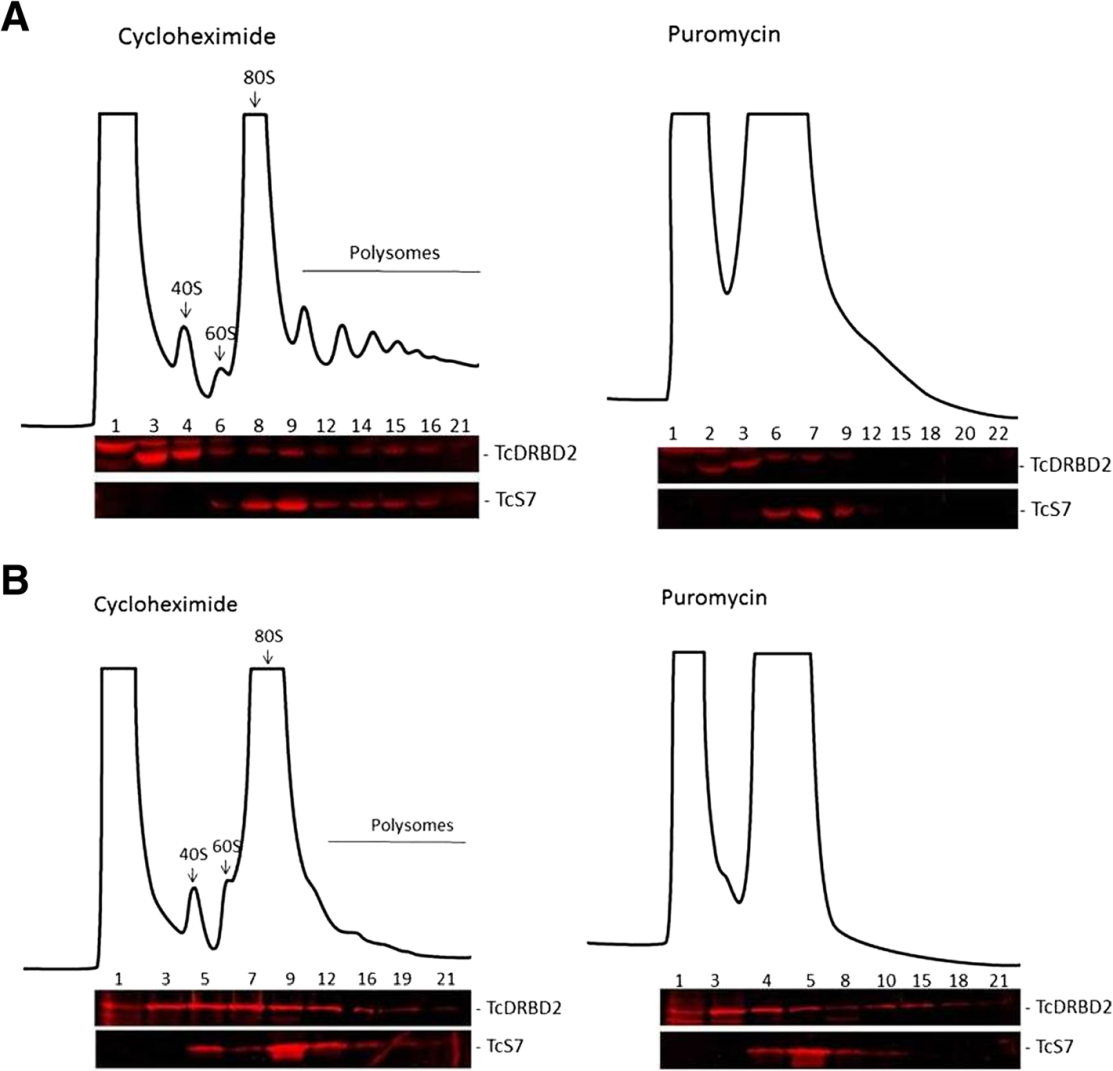
DRBD2 is part of translational complexes in unstressed epimastigotes. Polysome fractionation by sucrose density gradient. The fractions (1–22) were collected after the sedimentation of cytoplasmic lysates from epimastigotes (**a**) in exponential growth and (**b**) subjected to nutritional stress, both treated with 100 mg/ml cycloheximide or 2 mM puromycin. The 40S and 60S ribosomal subunits, the 80S ribosome monomer, and polysomes are indicated. Western blotting of alternate fractions was performed with anti-protein A antibody (1:40,000 dilution) for TAP-tagged DRBD2 (52 kDa) detection, and mouse anti-S7 (25 kDa) antibody (1:1000 dilution) for S7 detection, a ribosomal protein commonly used as a control

We investigated if DRBD2 was present in cytoplasmic complexes involved in translation by performing a polysome fractionation of epimastigotes in exponential growth (Fig. 3a) and epimastigotes subjected to a nutritional stress (Fig. 3b). Nutritional stress is one of the key steps that triggers *T. cruzi* metacyclogenesis [35]. DRBD2 was detected in polysome-enriched and polysome-independent fractions in both conditions analyzed - unstressed and stressed epimastigotes (Fig. 3a and b, respectively).

The sedimentation profile of DRBD2 on the sucrose density gradients of unstressed epimastigotes showed that its migration was similar to the S7 ribosomal protein - used as a control of polysomal fractions - after treatment with both cycloheximide and puromycin (Fig. 3a). These results indicate that DRBD2 is, in fact, associated with the translation machinery in unstressed epimastigotes. In stressed parasites, however, DRBD2 was associated with polysome-independent heavy complexes after puromycin treatment, differently from the control S7 (Fig. 3b), indicating that DRBD2 is not associated with the translation machinery in epimastigotes subjected to nutritional stress.

In yeast, Gbp2 is also present in cytoplasmic mRNP complexes formed by Gbp2, Npl3, and Hrbd1 [29]; this complex represses the translation of the regulated transcripts. During glucose deprivation, Gbp2, Npl3, and Hrbd1 were found in P-bodies and stress granules in yeast [36, 37], suggesting a translation arrest of the associated transcripts in this condition.

### DRBD2 associates with proteins involved in RNA metabolism in epimastigotes

**Fig. 4:**
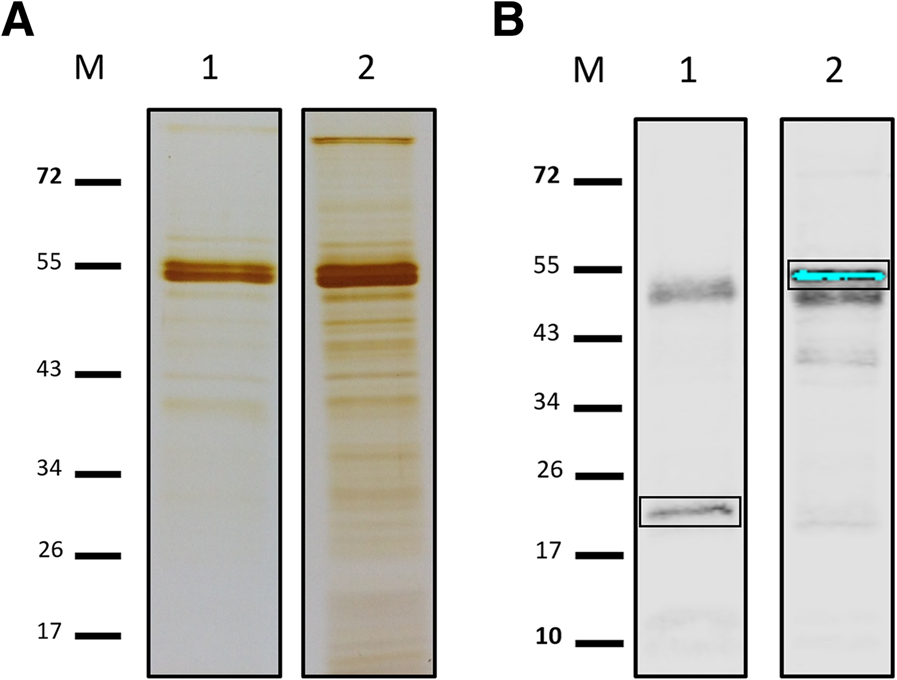
DRBD2 immunoprecipitation assay. **a** SDS-PAGE stained by silver nitrate of the eluted fractions of (1) the TAP-tagged control and (2) TAP-tagged DRBD2 IP assays. **b** Detection by western blotting of (1) TAP-tagged control and (2) TAP-tagged DRBD2 in the eluted fractions of the IP assays. Protein detection is indicated in boxes. TAP-tagged DRBD2 (52 kDa) and the TAP-tagged control (21 kDa) were detected by primary rabbit anti-protein A antibody (1:40,000 dilution). Bound antibodies were detected by the alkaline phosphatase method. M: PageRuler prestained Protein Ladder (Thermo Fisher Scientific). The additional protein bands detected in the membrane, at approximately 55 kDa, refer to the IgG chains from the magnetic beads used in the IP assay

The association of DRBD2 with translation complexes in unstressed epimastigotes motivated us to investigate the proteins partners of its mRNP complex. For this, we used immunoprecipitation (IP) assays and analyzed them by shotgun proteomics. Initially, we performed an SDS-PAGE of the IP assay eluates to compare DRBD2 immunoprecipitated complexes with the control, i.e., parasites expressing only TAP-tagging empty vector (Fig. 4). Figure 4a shows the detection of differentially abundant proteins in the DRBD2 immunoprecipitated complex compared to the control IP assay (lanes 2 and 1, respectively).

The complete proteomic results are presented in Additional file 4: Table S1. Tables 1 and 2 shortlist manually selected results based on abundancy (approximated by NIAF) and having a counterpart with an annotation in *T. brucei.* These proteins are involved in different processes of gene expression regulation and RNA metabolism in trypanosomatids, such as the RBPs ALBA1, ALBA3 and ALBA4, UBP1 and UBP2, DRBD3, and the poly(A) binding protein PABP2. In addition, we also identified a number of ribosomal proteins, reinforcing the previous observation, from the polysome fractionation, indicating that DRBD2 might be associated to translation regulation (Additional file 4: Table S1).

**Table 1:** Highlighted unique proteins identified in the DRBD2-mRNP complex. Unique proteins identified only in the TAP-tagged DRBD2 IP assay and not in the control. TriTrypDB ID and protein description: identification number and *T. cruzi* protein description from TriTryp database. Signal: sum of PatternLab’s normalized label-free quantitation derived from the extracted ion chromatograms - XIC; *T. brucei* protein description: the corresponding protein description of *T. brucei* from TriTrypDB

TriTrypDB ID	Signal	Protein description from TriTrypDB	*T. brucei* protein description
Tc00.1047053511817.180	0,008204787	Histone H2A	
Tc00.1047053510755.120	0,00780386	RNA-binding protein	DRBD2
Tc00.1047053511727.290	0,000890944	RNA-binding protein	NRBD1
Tc00.1047053510359.270	0,000837085	Mitochondrial RNA-binding protein 2	
Tc00.1047053510877.40	0,000762341	Uncharacterized protein	ALBA3
Tc00.1047053510877.30	0,000749083	Uncharacterized protein	ALBA4
Tc00.1047053506649.80	0,000495344	RNA-binding protein	DRBD3
Tc00.1047053504001.10	0,000476768	Uncharacterized protein	ALBA1
Tc00.1047053511285.120	0,000428502	ATP-dependent RNA helicase	Hel67
Tc00.1047053508895.60	0,000322697	RNA-binding protein	ZC3H40
Tc00.1047053511367.220	0,000274944	Uncharacterized protein	Ntf2-like
Tc00.1047053507093.229	0,000221601	U-rich RNA-binding protein UBP-2	UBP2
Tc00.1047053507093.220	0,000164223	RNA-binding protein	UBP1

**Table 2:** Highlighted enriched proteins in the DRBD2-mRNP complex. Enriched proteins identified in TAP-tagged DRBD2 and control IP assays with differential abundance. TriTrypDB ID and protein description: TriTryp database identification number and description for a given protein; Signal control and Signal DRBD2: PatternLab’s Normalized Ion Abundance Factors (NIAF) from control and DRBD2 replicates; Proteins with fold change > 2 were considered; Product: a given name for the corresponding protein

TriTrypDB ID	Signal control	Signal DRBD2	Fold change	Protein description from TriTrypDB	Product
Tc00.1047053508461.140	2,82E-05	0,004717835	167,14	Poly(A)-binding protein, putative	PABP2
Tc00.1047053510679.40	4,83E-05	0,001303308	26,99	Uncharacterized protein	
Tc00.1047053506959.30	8,23E-05	0,001759715	21,38	ATP-dependent DEAD/H RNA helicase	DHH1
Tc00.1047053511635.20	0,00046701	0,005277732	11,3	Histone H2B	
Tc00.1047053510769.49	0,000161566	0,001579402	9,78	40S ribosomal protein S6	
Tc00.1047053509247.30	6,94E-06	5,86E-05	8,44	Uncharacterized protein	
Tc00.1047053508177.90	8,26E-06	6,50E-05	7,87	Uncharacterized protein	
Tc00.1047053507831.60	4,00E-05	0,000298381	7,46	Heat shock protein, putative	
Tc00.1047053509695.170	0,00046333	0,002714777	5,86	60S ribosomal protein L9, putative	
Tc00.1047053510119.20	0,00297696	0,017012608	5,71	Elongation factor 1-alpha	EF1-alfa
Tc00.1047053510963.90	0,00285562	0,011081836	3,88	Elongation factor 2, putative	EF-2
Tc00.1047053510155.180	0,001556005	0,004652216	2,99	Eukaryotic initiation factor 4a	eIF4A

### Assessment of transcripts regulated by the DRBD2-mRNP complex

**Fig. 5:**
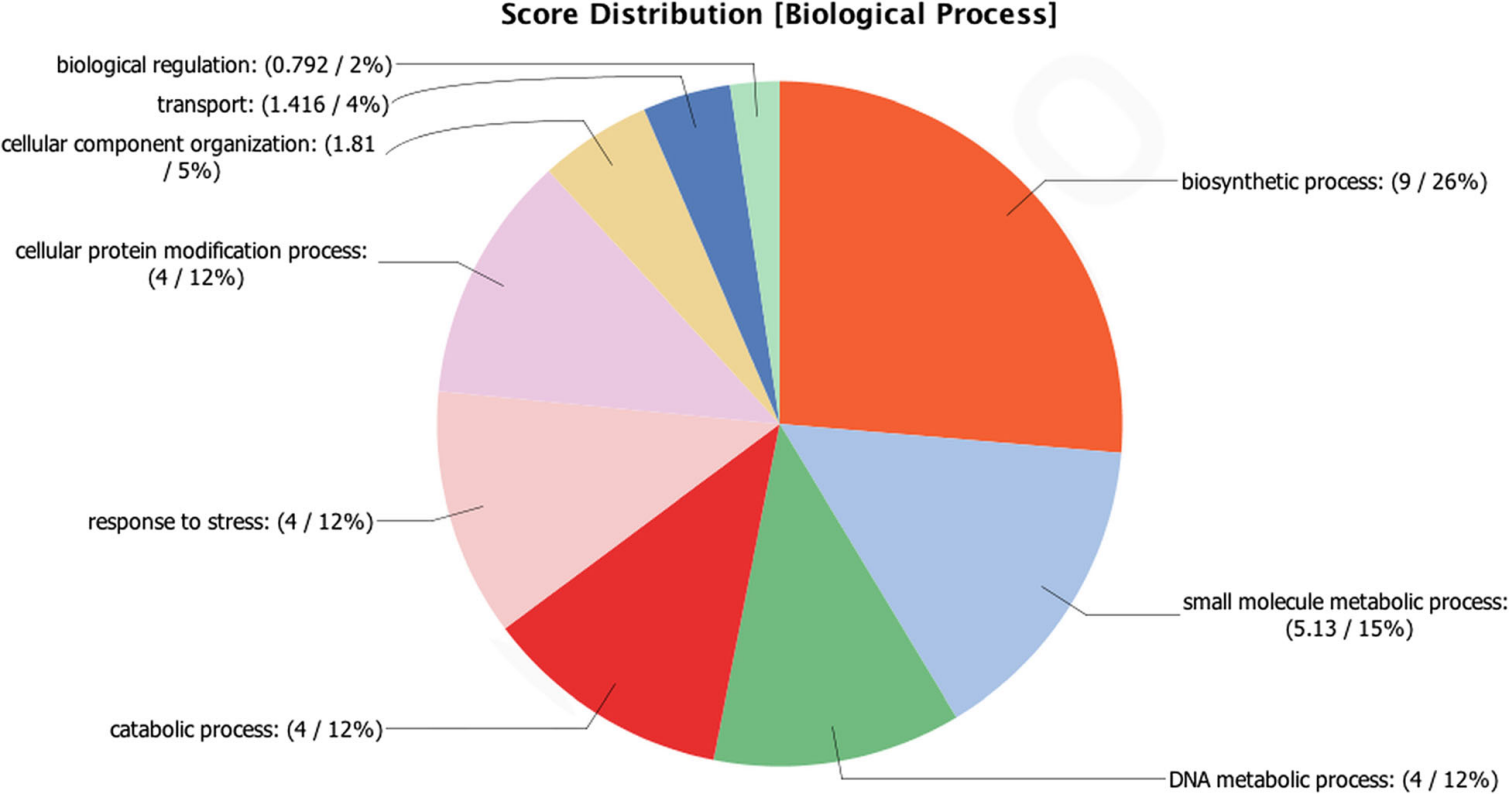
Gene ontology classification for biological processes of the identified transcripts of the DRBD2-mRNP complex. The numbers indicate the score of each gene ontology term and the percentage of each term in the group of transcripts being analyzed

We performed RNA-seq analysis of the immunoprecipitated DRBD2-mRNP complex to assess the regulated transcripts. The analysis pinpointed 86 transcripts as more abundant in the DRBD2-mRNP in epimastigotes when compared to the TAP-tag strain control, with an absolute fold-change of at least two (Additional file 5: Table S2 and Additional file 7: Table S4); however, most transcripts mapped to hypothetical proteins. To obtain further information regarding the transcripts, we performed gene ontology analysis (Fig. 5). Most of the mRNAs code for proteins related to biosynthetic process, DNA metabolic process, protein modification, and response to stress (Fig. 5). The complete information of the RNA-seq analysis is found in Additional file 5: Table S2, Additional file 6: Table S3, Additional file 7: Table S4. Table 3 lists the most abundant transcripts identified in this work.

**Table 3:** The most abundant transcripts associated with the DRBD2-mRNP complexes in epimastigotes. Gene ID: *T. cruzi* Dm28c strain identification number from the TriTryp database; Product description: resulting protein description provided by TriTrypDB

Gene ID	Product description	Max group mean	Log_2_ fold change	Fold change	FDR *p*-value
TCDM_06351	ATP-dependent DEAD/H DNA helicase recQ	16,788,12	12,33	5145,66	0%
TCDM_14363	hypothetical protein	1629,85	7,63	198,67	0%
TCDM_09386	hypothetical protein	929,52	7,41	169,66	0%
TCDM_01670	hypothetical protein	605,60	7,25	152,51	0%
TCDM_14159	hypothetical protein	192,69	6,88	118,06	0%
TCDM_01210	hypothetical protein	516,81	6,88	117,47	0%
TCDM_13811	hypothetical protein	386,65	6,72	105,38	0%
TCDM_09098	hypothetical protein	1242,50	6,53	92,72	1%
TCDM_02352	RNA-binding protein	695,00	6,18	72,45	0%
TCDM_06973	hypothetical protein	502,23	5,86	57,96	0%
TCDM_12750	trans-sialidase	47,34	5,86	57,94	0%
TCDM_12372	hypothetical protein	30,65	5,63	49,67	0%
TCDM_13701	unspecified product	347,66	5,54	46,46	1%
TCDM_05675	unspecified product	284,03	5,33	40,18	1%
TCDM_03204	hypothetical protein	323,38	5,21	36,91	1%
TCDM_02335	UDP-galactose transporter	190,16	4,84	28,71	1%
TCDM_05198	DNA polymerase delta catalytic subunit	53,35	4,80	27,90	0%
TCDM_12807	hypothetical protein	58,72	4,75	26,88	1%
TCDM_00712	hypothetical protein	101,03	4,59	24,14	0%
TCDM_09723	hypothetical protein	94,04	4,53	23,03	1%
TCDM_03727	hypothetical protein	34,10	4,50	22,55	0%
TCDM_13534	hypothetical protein	9,56	4,35	20,41	0%
TCDM_06859	hypothetical protein	167,14	4,24	18,86	1%
TCDM_08048	hypothetical protein	13,59	4,01	16,15	0%
TCDM_06310	quiescin sulfhydryl oxidase	7,16	3,95	15,47	0%
TCDM_13669	hypothetical protein	7,50	3,95	15,45	0%
TCDM_14079	hypothetical protein	27,07	3,93	15,28	0%
TCDM_13037	hypothetical protein	79,99	3,90	14,93	0%
TCDM_03172	small GTP-binding protein RAB6	53,57	3,82	14,17	1%
TCDM_04048	hypothetical protein	49,91	3,75	13,50	0%
TCDM_11018	P27 protein	117,38	3,72	13,22	0%
TCDM_00934	hypothetical protein	37,36	3,72	13,17	1%
TCDM_02364	hypothetical protein	87,49	3,71	13,11	0%
TCDM_03710	nuclear lim interactor-interacting factor	19,47	3,68	12,84	0%
TCDM_03688	ATP-dependent DEAD/H RNA helicase - DHH1	39,78	3,68	12,77	0%
TCDM_10796	ATPase	34,11	3,66	12,65	1%
TCDM_12205	complement regulatory protein	12,93	3,62	12,27	1%
TCDM_07935	hypothetical protein	6,67	3,61	12,22	1%
TCDM_13524	hypothetical protein	55,72	3,54	11,66	1%
TCDM_14171	hypothetical protein	79,57	3,53	11,55	1%
TCDM_06468	hypothetical protein	11,22	3,50	11,35	1%
TCDM_01286	hypothetical protein	50,27	3,48	11,17	1%
TCDM_05765	DNA-directed RNA polymerase III subunit	34,71	3,47	11,06	1%
TCDM_03285	small GTP-binding protein Rab11	123,34	3,42	10,74	1%
TCDM_09209	fructosamine kinase	41,17	3,38	10,42	1%
TCDM_13613	retrotransposon hot spot (RHS) protein	10,02	3,36	10,26	0%
TCDM_10239	hypothetical protein	60,17	3,35	10,18	1%
TCDM_14224	hypothetical protein	172,44	3,32	10,00	1%

We then used the results from the *T. cruzi* ribosome profiling by Smircich et al. [34] to verify if the transcripts identified in this work are associated with polysomes (Additional file 5: Table S2 and Additional file 7: Table S4). Interestingly, some of the most abundant transcripts associated with the DRBD2-mRNP complex showed high levels of expression in the total RNA fraction rather than in the polysomes, in both epimastigotes and metacyclic trypomastigotes (Additional file 3: Figure S3C), indicating a possible role of DRBD2 in negatively regulating the translation of its target transcripts, as described in *T. brucei* [25]*.*

## Discussion

RBPs play lead roles in gene expression regulation in trypanosomatids [10]. The increasing knowledge about the molecular functioning of these proteins in *T. cruzi* has helped us to gradually understand how the parasite copes to survive facing various biological diversities throughout its life cycle [38].

Here, we studied the RBP DRBD2 in *T. cruzi*, an ortholog of the SR-rich protein Gbp2 in yeast, which forms a complex with Hrbd1 and Npl3 to act in the quality control and export of nascent mRNAs [24, 29]. DRBD2 is a cytoplasmic protein that shifts its cellular localization to a granular pattern when subjected to a nutritional stress. Additionally, we showed that DRBD2 participates in translational complexes in epimastigotes, according to its sedimentation profile on a sucrose gradient. The shotgun proteomic analysis identified mostly proteins involved in RNA metabolism and regulation; some examples are ZC3H39, UBP1, UBP2, ALBA3, ALBA4, and NRBD1.

In *Leishmania,* ALBA1, 3 and 4 coimmunoprecipitate with DRBD2 in amastigotes [23]; Ntf2-like and PABP2 also associate with ALBA proteins, suggesting that a protein complex is formed by these partners. These results are consistent with our results; PABP2, Ntf2-like, ALBA1, 3, and 4 were also detected in the DRBD2-mRNP complex in *T. cruzi*. Moreover, ALBA proteins are associated with the translation machinery in *T. brucei* [21, 22], such as DRBD2 in *T. cruzi*. When *T. brucei* parasites were subjected to a nutritional stress, ALBA3 and ALBA4 associated with DHH1 to form cytoplasmic stress granules, resulting in a reduced translation rate [22]. Interestingly, we observed that DHH1, ALBA3, and ALBA4 associate with the DRBD2-mRNP complex.

In *T. brucei*, DRBD3 is an RBP that regulates transcripts of ribosomal proteins and translation factors and is thus involved in translation control [19]. Furthermore, TbDRBD3 is part of a cytoplasmic mRNP complex with PABP2 and Ntf2-like [26], which is consistent with our results (Tables 1 and 2). Interestingly, Hel67 was also associated with the TcDRBD2 complex; in *Leishmania*, Hel67 is a cytoplasmic ATP-dependent DEAD-box helicase that binds to specific ribosomal RNAs, preventing its degradation and ultimately inhibiting cell death [39].

The poly(A)-binding protein PABP2 is highly abundant in the DRBD2 complex (Additional file 4: Table S1). In *T. brucei*, PABP2 may accumulate in nuclear periphery granules, most likely regulating the nuclear export of transcripts [40]. TbDRBD2 is also present in the TbPABP2 complex, together with TbZC3h40, TbALBA1, and TbALBA3 [41]; these results are consistent with ours (Tables 1 and 2).

Finally, the elongation factor 1 alpha (EF-1α) is also present in the DRBD2-mRNP complex. This protein is conserved in eukaryotic organisms [42]; it is a typical moonlighting protein and is involved in cellular processes such as translation and RNA transport [43]. In *T. cruzi*, EF-1α is associated with both polysome and polysome-free fractions in epimastigotes 6; also, the EF-1α-mRNP complex seems to play a role in stress response by regulating different sets of transcripts in this condition [42]. In addition, ribosomal proteins are abundantly present in ribonucleoprotein (mRNP) complexes because they are usually involved in translation, hence they are considered an indicative of translation regulation by these complexes, as described for NRBD1 from *T. cruzi* [11].

The RNA-seq results showed that most of the transcripts regulated by the DRBD2 complex mapped to hypothetical proteins related to multiple processes, such as to biosynthetic process, DNA metabolic process, protein modification, and response to stress.

## Conclusion

RBPs are key proteins in gene expression regulation of *T. cruzi*. The information regarding the actors and mechanisms in this field are continually increasing. DRBD2’s orthology to yeast Gbp2 is a promising indicative of the similarity between these proteins roles in negatively regulating their targets in mRNP complexes. Moreover, DRBD2 seems to be involved in RNA metabolism regulation in *T. cruzi* once several regulatory proteins were identified by the shotgun proteomic analysis of the immunoprecipitated complexes.

We consider these results an important contribution to future studies regarding gene expression regulation in *T. cruzi*, especially in the field of RNA-binding proteins.

## Methods

### In silico analysis

Similarities between the DRBD2 (TcCLB.510755.120) sequence from *T. cruzi* and the Gbp2 sequences from several other organisms from NCBI Refseq and UniProt SwissProt (accessed in September, 2018), including *Trypanosoma brucei*, *Saccharomyces cerevisiae,* and *Homo sapiens,* were assessed using the BLAST algorithm [44]. This analysis was achieved by querying the TcDRBD2 sequence against other organism genomes, followed by inverse alignment using the best candidates from the first search, using the best reciprocal hit (BRH) method [45]. Plus, we used Constraint-based Multiple Alignment Tool (COBALT), contained in NCBI platform, to perform a multiple protein sequence alignment using conserved domain and local sequence similarity information. To predict phosphorylation sites in the DRBD2 sequence, we used the NetPhos 2.0 Server [46] (http://www.cbs.dtu.dk/services/NetPhos/). Secondary structure mapping was obtained with PSIPRED [47].

### Parasite cultures and drug treatments

*T. cruzi* Dm28c strain was originally isolated from *Didelphis marsupialis* [35, 48] and epimastigotes forms were maintained in axenic culture in liver infusion tryptose (LIT) medium at 28 °C [48]. To prepare 1.0 L of LIT medium, we used 9.0 g of liver infusion broth, 5.0 g of tryptose, 1.0 g of NaCl, 8.0 g of Na_2_HPO_4_, 0.4 g of KCl, 1.0 g of glucose, 100 ml of fetal bovine serum (heat-inactivated), 10 mg of hemin, and 1.0 L of distilled water. For nutritional stress induction, epimastigotes in the late logarithmic growth phase were harvested by centrifugation at 7000 x *g* for 5 min at 10 °C and incubated for 2 h at 28 °C in TAU medium (190 mM NaCl, 17 mM KCl, 2 mM MgCl_2_, 2 mM CaCl_2_, 8 mM phosphate buffer pH 6.0) at a density of 5.0 × 10^8^ parasites/ml. Metacyclic trypomastigotes were prepared as previously described [48].

### Recombinant protein expression

The *T. cruzi* Dm28c strain was used as the DNA template. The DRBD2 (TriTrypDB ID TcCLB.510755.120) open reading frame polymerase chain reaction (PCR) product was cloned into the pDONR™221 vector from Gateway Technology (Invitrogen) and was then recombined into a pTcTAPN vector that adds the Tap-tag in the N-terminus portion of the protein [49]. *T. cruzi* Dm28c epimastigotes were transfected with these plasmids as described by Lu et al. [50]. Stable lines were selected by adding 500 mg/ml G418 to the culture medium. It is episomally expressed, not recombining into a genomic locus (DRBD2 molecular weight: 31 kDa; TAP-tagging: 21 kDa).

### Immunoblotting and immunofluorescence

Immunoblotting and immunofluorescence were performed as previously described [51]. A rabbit anti-protein A antibody (Sigma-Aldrich) was used to detect TAP-tagging (1:40,000 dilution). TcDHH1 (47 kDa) was used as a control of the metacyclogenesis western blotting assay. Anti-TcDHH1 (1:100 dilution) was kindly provided by Jimena Ferreira da Costa. Bound antibodies were detected by the alkaline phosphatase method or by the Odyssey® imager (LI-COR) using the secondary antibodies IRDye 800CW or IRDye 680RD (1:15,000 dilution) [52]. Immunofluorescence slides were analyzed by inverted microscopy (Leica DMI6000B) associated with deconvolution software (Leica AF6000, microscope facility RPT07C PDTIS/ Carlos Chagas Institute - Fiocruz Paraná).

### Polysome sedimentation profiles

Polysomes were separated on a sucrose density gradient as previously described, performed once for each condition [53]. The drugs used here were puromycin, which causes premature chain termination during translation, thus disassembling polysomes [54]; and cycloheximide, that interferes with the translocation step in protein synthesis, thus blocking eukaryotic translational elongation and stabilizing polysomes [54]. The resulting fractions were analyzed by western blotting using rabbit anti-protein A (1:40,000 dilution) for DRBD2 detection and mouse anti-S7 (1:1000 dilution), kindly provided by Dr. Stênio P. Fragoso. S7 (25 kDa) is a small ribosomal protein commonly used as a control. The fractionation was performed twice for each treatment and condition.

### Immunoprecipitation assays

Immunoprecipitation (IP) assays were performed with cytoplasmic extracts of exponentially growing epimastigotes. Primary rabbit anti-protein A antibodies were coupled to 30 mg of Dynabeads® M-270 Epoxy (Thermo Fisher), following the manufacturer’s instructions. The equivalent of 5.0 × 10^9^ parasites expressing TAP-tagged DRBD2 and control parasites expressing only the TAP-tag were cryo-milled using the Mixer Mill MM 400 (Retsch), resulting in 1 g of cell powder. Approximately 5.0 × 10^8^ parasites were resuspended in 1 ml of solubilization buffer [25 mM and 50 mM sodium citrate; 20 mM Tris-HCl pH 7.5; 5 mM MgCl_2_; 0.1% CHAPS; protease inhibitor (Complete Mini Protease inhibitor cocktail tablet, Roche, 1:100) and 40 U/ml RNase OUT (Invitrogen)], followed by centrifugation at 20,000 x *g* for 10 min at 4 °C. The supernatant was incubated with 3 μl of the magnetic beads under constant stirring at 4 °C for 2 h. The magnetic beads were washed 3 times with the solubilization buffer, and the proteins linked to the beads were eluted with 30 μl of elution buffer (2% SDS and 20 mM Tris-HCl pH 8.0) at 72 °C for 20 min. The eluted fractions were visualized using 13% SDS-PAGE with silver nitrate staining, as previously described [55], and then, TAP-tagged DRBD2 was detected by immunoblotting.

### Shotgun proteomic data acquisition and analysis

Digested extracts of the immunoprecipitated lysates were fractionated by a NanoLC Easy 1000 system (Thermo Fisher Scientific). Ionized peptides were analyzed by an LTQ Orbitrap XL ETD (Thermo Fisher Scientific). Sequences of *T. cruzi* were downloaded from TriTrypDB on March 16, 2016. PatternLab for proteomics 4.0 was used for data analysis, following the provided instructions [56]. Briefly, the search was performed using PatternLab’s integrated Comet [57] search engine. The validity of the Peptide Sequence Matches (PSM) was assessed using PatternLab’s Search Engine Processor module (SEPro) [58]. A cutoff score was established to accept a false discovery rate (FDR) of 1% based on the number of decoys [59]. The results were postprocessed to accept PSM with less than 5 ppm from the average ppm of the pre-validated results. Relative quantitation was performed according to PatternLab’s Normalized Ion Abundance Factors (NIAF) as a relative quantitation strategy [56]. For proteins present in both conditions (DRBD2 assay and control), we used PatternLab’s TFold module [60], with an FDR of 0.05. Only proteins having an absolute fold change ≥ 2 were considered for the TFold analysis.

### Ribonomic data acquisition and analysis

The eluted fractions from the immunoprecipitation assays were purified with the miRCURY™ RNA Isolation Kit (Exiqon), with the “Cell & Plant” protocol from the manufacturer’s manual, initiating with the “Cell Lysis” step in the manual. The purified RNAs were quantified and analyzed using the Agilent DNA 1000 kit on an Agilent Technologies 2100 Bioanalyzer and then subjected to sequencing on the Illumina® platform using paired-end configuration on the MiSeq Desktop Sequencer (Illumina®). The sequencing data obtained from 3 biological replicates were analyzed using CLC Genomics Workbench© 7.5.5. The *T. cruzi* Dm28c reference genome used for mapping was obtained from the NCBI database (AYLP01), and the alignment was performed as follows: additional upstream and downstream sequences of 75 bases, minimum number of reads: 10, maximum number of mismatches: 2, and nonspecific match limit: - 2. We selected possible targets of the DRBD2-mRNP with the β binomial statistical test (Baggerly’s test [61]) with a *q*-value ≤1%, a minimum RPKM value of 50 and a fold change > 5 when compared to the control (parasites expressing TAP-tag) for significance. The RNA-seq data were deposited in the NCBI Sequence Read Archive (SRA) database under the accession number SRX4560510.

## Additional files

Additional file 1: **Figure S1.** DRBD2 is an ortholog of yeast Gbp2. Protein alignment based on NCBI Constraint-based Multiple Alignment Tool (COBALT); this tool performs multiple protein sequence alignment using conserved domain and local sequence similarity information. Red bars indicate the regions of proteins sequences similarities. (TIFF 781 kb)

Additional file 2: **Figure S2.** Phosphorylation sites predicted by NetPhos 2.0 server. DRBD2 sequence provided by TriTrypDB with 276 amino acids. DRBD2 amino acid sequence and DRBD2 predicted phosphorylation sites. Ser: serine; Thr: threonine; Tyr: tyrosine. (TIF 54 kb)

Additional file 3: **Figure S3.** Expression levels of *drbd2* transcript and the targets of DRBD2-mRNP. (A) *drbd2* expression level in the developmental forms (data from Li et al., 2016 [33] available at tritrypdb). (B) *drbd2* expression level in the epimastigotes and trypomastigote forms - RNA-seq (total RNA) and ribosome profiling sequencing (Ribo-seq) (data from Smircich et al., 2015 [34] aviailable at tritrypdb). (C) Expression levels of the most abundant transcripts associated to DRBD2-mRNP complex in the epimastigotes and trypomastigote forms - RNA-seq (Total RNA) and ribosome profiling sequencing (Ribo-seq) (data from Smircich et al., 2015 aviailable at tritrypdb). In the X-axis the expression levels in FPKM (Fragments Per Kilobase Million). (TIFF 385 kb)

Additional file 4: **Table S1.** Complete information of the shotgun proteomic analysis of the DRBD2-mRNP complex. Unique proteins: proteins identified only in DRBD2 IP assays. Signal: sum of PatternLab’s normalized label-free quantitation derived from the extracted ion chromatograms – XIC; Protein description and ID from TriTrypDB: TriTryp database description and identification number for a given protein. Product: the protein given name from TriTrypDB. Enriched proteins: proteins identified in DRBD2 IP and control IP replicates with differential abundance. Proteins with fold change ≥2 were considered. Signal DRBD2 and Signal control: signal intensity of the proteins in DRBD2 IP and control IP replicates, respectively. (XLSX 38 kb)

Additional file 5: **Table S2.** Transcripts identified by the ribonomic analysis of the DRBD2-mRNP complex. Transcripts identified by RNA-seq from the DRBD2-mRNP complex from epimastigotes in exponential growth. The table also shows the transcripts regulated by DRBD2 that are associated with polysomes in epimastigotes and metacyclic trypomastigotes, information provided by Smircich et al. (XLSX 224 kb)

Additional file 6: **Table S3.** DRBD2-mRNP complex RNA-seq mapping statistics information. (XLSX 5 kb)

Additional file 7: **Table S4.** Complete information of the ribonomic analysis of the DRBD2-mRNP complex. (XLSX 4213 kb)

## Data Availability

The RNA-seq data were deposited in the NCBI Sequence Read Archive (SRA) database under the accession number SRX4560510. The proteomic raw data is available at http://proteomics.fiocruz.br/supplementaryfiles/wippel2018/.

## Abbreviations

ARE: AU-rich elements
FDR: False discovery rate
IP: Immunoprecipitation
LIT: Liver infusion tryptose
mRNA: messenger RNA
mRNP: ribonucleoprotein
NIAF: Normalized Ion Abundance Factors
PCR: Polymerase chain reaction
PSM: Peptide Sequence Matches
RBP: RNA-binding proteins
RRM: RNA Recognition Motif
SEPro: Search Engine Processor
SR: Serine-arginine
SRA: Sequence Read Archive
