# Supplementary figures and images for: Unveiling the partners of the DRBD2-mRNP complex, an RBP in Trypanosoma cruzi and ortholog to the yeast SR-protein Gbp2
Source: BMC Microbiol. 2019 Jun 11;19:128. doi: 10.1186/s12866-019-1505-8 (PMC6560856; doi:10.1186/s12866-019-1505-8)

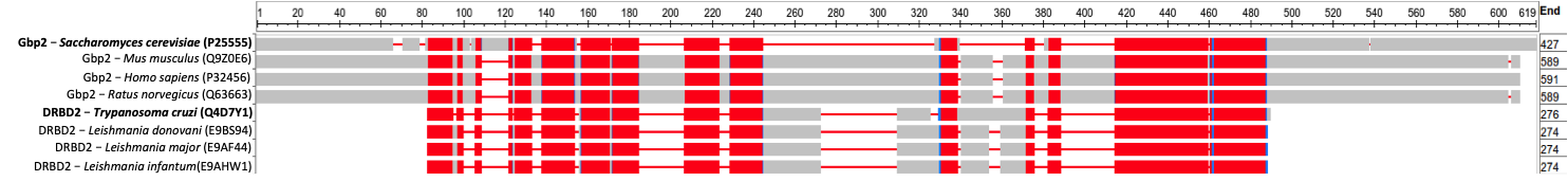

Supplement: Supplementary file 1 — Figure S1. DRBD2 is an ortholog of yeast Gbp2. Protein alignment based on NCBI Constraint-based Multiple Alignment Tool (COBALT); this tool performs multiple protein sequence alignment using conserved domain and local sequence similarity information. Red bars indicate the regions of proteins sequences similarities. (TIFF 781 kb) [file 12866_2019_1505_MOESM1_ESM.tiff]

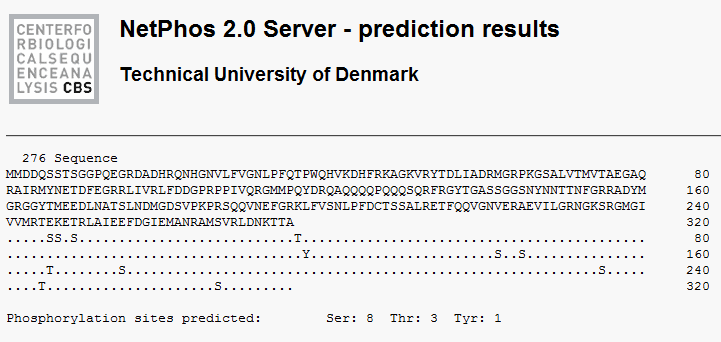

Supplement: Supplementary file 2 — Figure S2. Phosphorylation sites predicted by NetPhos 2.0 server. DRBD2 sequence provided by TriTrypDB with 276 amino acids. DRBD2 amino acid sequence and DRBD2 predicted phosphorylation sites. Ser: serine; Thr: threonine; Tyr: tyrosine. (TIF 54 kb) [file 12866_2019_1505_MOESM2_ESM.tif]

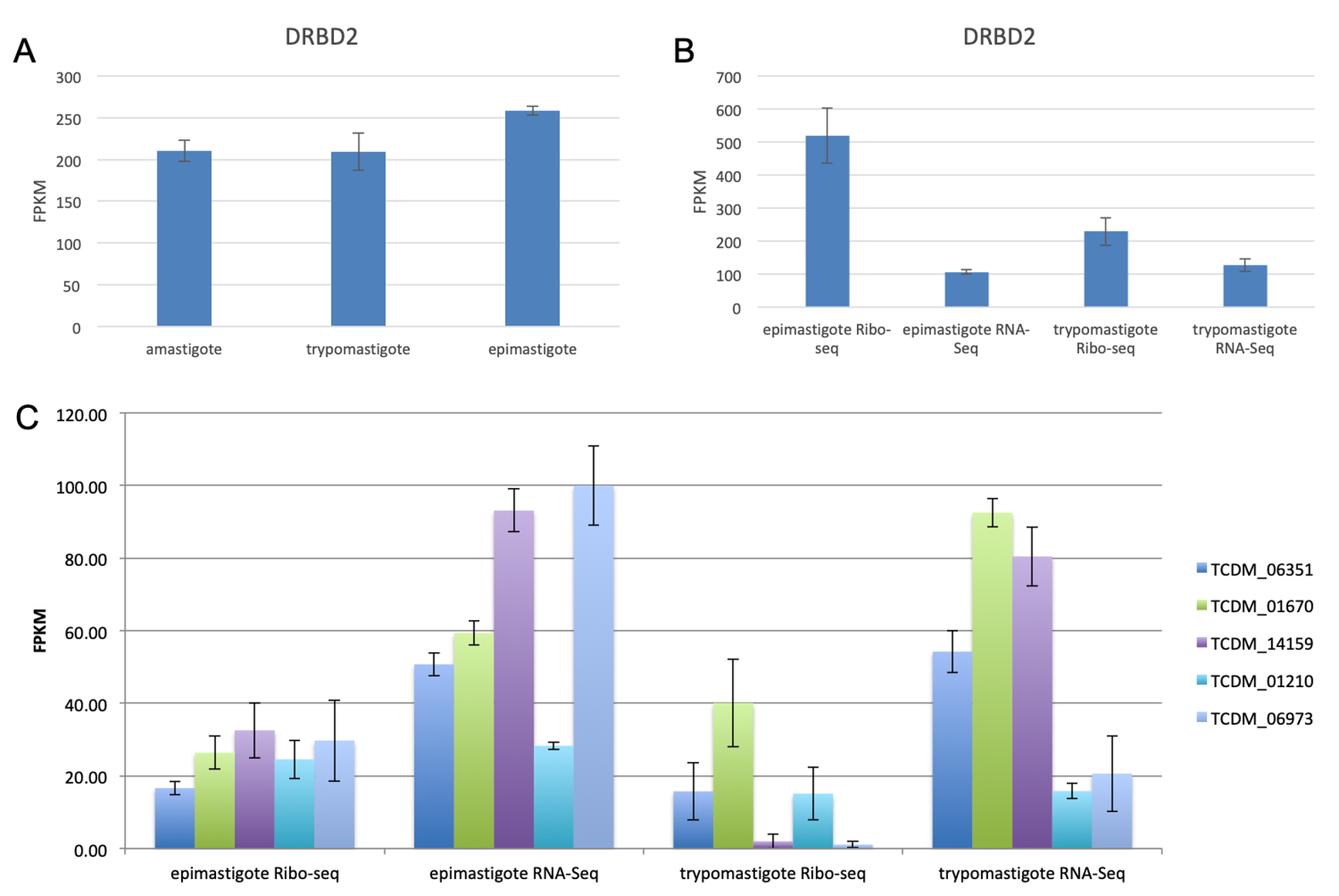

Supplement: Supplementary file 3 — Figure S3. Expression levels of drbd2 transcript and the targets of DRBD2-mRNP. (A) drbd2 expression level in the developmental forms (data from Li et al., 2016 [33] available at tritrypdb). (B) drbd2 expression level in the epimastigotes and trypomastigote forms - RNA-seq (total RNA) and ribosome profiling sequencing (Ribo-seq) (data from Smircich et al., 2015 [34] aviailable at tritrypdb). (C) Expression levels of the most abundant transcripts associated to DRBD2-mRNP complex in the epimastigotes and trypomastigote forms - RNA-seq (Total RNA) and ribosome profiling sequencing (Ribo-seq) (data from Smircich et al., 2015 aviailable at tritrypdb). In the X-axis the expression levels in FPKM (Fragments Per Kilobase Million). (TIFF 385 kb) [file 12866_2019_1505_MOESM3_ESM.tiff]
